# Microvascular permeability during experimental human endotoxemia: an open intervention study

**DOI:** 10.1186/cc3050

**Published:** 2005-02-21

**Authors:** Lucas TGJ van Eijk, Peter Pickkers, Paul Smits, Wim van den Broek, Martijn PWJM Bouw, Johannes G van der Hoeven

**Affiliations:** 1 Departments of Intensive Care Medicine and Pharmacology-Toxicology, Radboud University Nijmegen Medical Centre, Nijmegen, The Netherlands; 2 Department of Pharmacology-Toxicology, Radboud University Nijmegen Medical Centre, Nijmegen, The Netherlands; 3 Department of Nuclear Medicine, Radboud University Nijmegen Medical Centre, Nijmegen, The Netherlands; 4 Department of Intensive Care Medicine and Nijmegen UniversityCenter for Infectious Diseases, Radboud University Nijmegen Medical Centre, Nijmegen, The Netherlands

## Abstract

**Introduction:**

Septic shock is associated with increased microvascular permeability. As a model for study of the pathophysiology of sepsis, endotoxin administration to humans has facilitated research into inflammation, coagulation and cardiovascular effects. The present study was undertaken to determine whether endotoxin administration to human volunteers can be used as a model to study the sepsis-associated increase in microvascular permeability.

**Methods:**

In an open intervention study conducted in a university medical centre, 16 healthy volunteers were evaluated in the research unit of the intensive care unit. Eight were administered endotoxin intravenously (2 ng/kg *Escherichia coli *O113) and eight served as control individuals. Microvascular permeability was assessed before and 5 hours after the administration of endotoxin (*n *= 8) or placebo (*n *= 8) by three different methods: transcapillary escape rate of I^125^-albumin; venous occlusion strain-gauge plethysmography to determine the filtration capacity; and bioelectrical impedance analysis to determine the extracellular and total body water.

**Results:**

Administration of endotoxin resulted in the expected increases in proinflammatory cytokines, temperature, flu-like symptoms and cardiovascular changes. All changes were significantly different from those in the control group. In the endotoxin group all microvascular permeability parameters remained unchanged from baseline: transcapillary escape rate of I^125^-albumin changed from 7.2 ± 0.6 to 7.7 ± 0.9%/hour; filtration capacity changed from 5.0 ± 0.3 to 4.2 ± 0.4 ml/min per 100 ml mmHg × 10^-3^; and extracellular/total body water changed from 0.42 ± 0.01 to 0.40 ± 0.01 l/l (all differences not significant).

**Conclusion:**

Although experimental human endotoxaemia is frequently used as a model to study sepsis-associated pathophysiology, an endotoxin-induced increase in microvascular permeability *in vivo *could not be detected using three different methods. Endotoxin administration to human volunteers is not suitable as a model in which to study changes in microvascular permeability.

## Introduction

Sepsis is the leading cause of mortality in noncardiac intensive care units, resulting in an estimated mortality of 200,000 patients per year in the USA alone [1]. Sepsis is notably characterized by an increase in microvascular permeability, which accounts for the extravasation of macromolecules and fluid from the plasma to the tissues. The impaired diffusion of oxygen to cells as a result of the extracellular oedema appears to be a critical factor in the development of multiple organ failure [2,3]. Few studies have been conducted in humans to examine the mechanism that underlies the sepsis-associated increase in microvascular permeability.

Endotoxin is among the principal bacterial components that interacts with the host during Gram-negative sepsis [4]. Administration of endotoxin to humans is an appropriate model in which to investigate acute inflammatory responses (activation of cytokines and coagulation pathways) and to evaluate novel therapeutic interventions [5]. *In vitro*, exposure of human endothelial cells to endotoxin induces an increase in permeability [6], and *in vivo *an increase in microvascular permeability is among the major manifestations observed in animal endotoxaemia [7-12]. In humans, microvascular permeability can be assessed by plasma disappearance of a tracer (e.g. I^125^-albumin), changes in tissue volume caused by an imposed hydrostatic pressure and changes in bio-impedance. These methods were validated for the detection of a modest increase in microvascular permeability in patients with various diseases [13-17] and, more relevant to our study, patients with sepsis or septic shock [18-20]. In septic patients, transcapillary escape rate of albumin varies from 6.7%/hour [21] to 13.4%/hour [18], whereas permeability measured using venous congestion plethysmography (VCP) ranged from 6.1 ml/min per 100 ml mmHg × 10^-3 ^[19] to 9.3 ml/min per 100 ml mmHg × 10^-3 ^[22].

The present study was undertaken to determine whether endotoxin administration to human volunteers can be employed as a model in which to study the sepsis-associated increase in microvascular permeability.

## Materials and methods

### Subjects

After approval had been granted by the local ethics committee, 16 nonsmoking individuals gave written informed consent to participate in the study. Those who were taking prescription drugs or asprin or other nonsteroidal anti-inflammatory drugs were excluded (except for oral anticontraceptives). Screening of the participants before the test revealed no abnormalities in medical history or physical examination. Routine laboratory tests and electrocardiograms were normal. All participants were HIV and hepatitis B negative. They had not suffered a febrile illness within the 2 weeks preceding the study. Ten hours before the experiment, the participants refrained from consuming caffeine, alcohol and food.

### Study design and procedures

Heart rate was continuously monitored using a three-lead electrogradiograph. An intra-arterial catheter in the radial artery permitted arterial blood sampling as well as continuous monitoring of blood pressure throughout the experiment. Forearm blood flow was measured in both arms using VCP, as described previously [23]. All participant received an intravenous infusion of a glucose/saline solution (2.5% glucose, 0.45% saline; 75 cm^3^/hour) via a cannula in an antecubital vein. At baseline, purified lipopolysaccharide (LPS) prepared from *Escherichia coli *O113 was injected intravenously (2 ng/kg) over 1–2 min in eight individuals, followed by 5 ml normal saline to ensure complete delivery. Another eight served as control individuals and received NaCl 0.9% instead of endotoxin in an equivalent volume. Because of obvious symptomatic changes after infusion of endotoxin, neither the volunteers nor the staff members were blinded to the study protocol.

The course over time of temperature, C-reactive protein, and plasma levels of tumour necrosis factor (TNF)-α and interleukin (IL)-1β [24] were monitored to confirm the inflammatory effects of endotoxin administration.

### Transcapillary escape rate of I125albumin

Microvascular permeability determined by the transcapillary escape rate of I^125^-albumin (TER-alb) was measured at baseline and 5 hours after endotoxin administration, when haemodynamic changes are at their maximum [25]. I^125 ^labelled albumin solution of 2 μCi (baseline) and 6 μCi (at 5 hours) in 5 cm^3 ^normal saline were given as an intravenous bolus injection followed by 5 cm^3 ^normal saline. The second dose is higher to overcome the background signal of the first dose. Arterial blood samples were drawn at baseline, and at 5, 10, 15, 20, 30, 45 and 60 min. Plasma radioactivity was measured in each sample using a scintillation detector (automatic γ-counter; 1480 Wizard 3", Wallac, Turku, Finland).

### Venous congestion plethysmography

Microvascular permeability was also determined by VCP, in accordance with methods fully described previously [26,27]. Microvascular filtration capacity (K_f_) – an index of vascular permeability – was measured using a protocol in which a series of eight small (10 mmHg) cumulative pressure steps were applied to venous congestion cuffs placed around both upper arms. K_f _was estimated from alterations in forearm circumference due to the pressures applied, using the Filtrass strain gauge plethysmograph (Filtrass Angio, DOMED, Munich, Germany) [27]. Using this system, no change in the recorded signal is observed until ambient venous pressure in the arm is exceeded. At congestion cuff pressures greater than this value, each additional pressure increment causes a change in forearm volume that is attributed to vascular filling. When the congestion cuff pressure exceeds the isovolumetric venous pressure, a steady state change in volume is observed, reflecting fluid filtration. K_f _reflects the product of the area available for fluid filtration and the permeability per unit surface area. Computer-based analysis enables differentiation between volume and filtration responses [28]. The value of K_f _is determined by linear regression of the fluid filtration as a function of the cuff pressure. The slope of this relationship is K_f _and the units are expressed as K_f_U (ml/min per 100 ml mmHg × 10^-3^) [28]. The files were recorded and saved for subsequent offline analysis. K_f _measurements were conducted before, and 4.5 hours and 22 hours after the administration of endotoxin or normal saline.

### Bioelectrical impedance analysis

In septic patients, fluid shifts from intracellular water to extracellular water (ECW) and an increase in total body water (TBW) occur because of an altered cellular membrane function, resulting in the formation of oedema. Bioelectrical impedance analysis (BIA) can estimate body composition parameters and has been used to estimate body water distribution and cellular membrane function in healthy individuals [29] and intensive care patients [20,30-33]. The principles of bioelectrical impedance postulate that resistance (R) is the opposition of TBW and electrolytes to the flow of an alternating current of low amplitude (800 μA) and high frequency (50 kHz). Reactance is the capacitance produced by tissue interfaces and cell membranes. An increase in microvascular permeability and an altered membrane function result in the formation of oedema, which decreases the resistance and reactance to an alternating electric current throughout the body. ECW will increase in relation to TBW, and reactance/resistance will decrease. BIA was performed using a body composition analyzer (Akern Srl, Florence, Italy). This device employs four-electrode polarization and measures the resistance and reactance of a conductor to application of an alternating electric current of 800 μA and 50 kHz. All measurements were made with the patient supine, with their arms relaxed at their sides but not touching their body, and with their thighs slightly separated. Electrodes were placed on the dorsal surface of the skin of the wrist and ankle, with the detector electrodes applied along the articulation bisecting line of both joints. BIA was performed at baseline and 4, 6, 8 and 22 hours after endotoxin administration.

### Drugs and solutions

All solutions were freshly prepared on the day of the experiment. Endotoxin from *Escherichia coli *(batch 0:113, lot G2B274) was obtained from US Pharmacopia Convention (Rockville, MD, USA) and dissolved in normal saline 0.9% to a concentration of 200 EU/ml (0.1 ml/kg). I125-albumin (Iodinated [125I] Human Serum Albumin; code IM 17 P) was obtained from Amersham International (Amersham, UK).

### Data analysis, calculations and statistics

Power analysis was based on clinically relevant changes in TER-Alb. In a previous study using the TER-alb method, we found a standard deviation ranging from 1.5% to 2.5%. An increase in transcapillary escape rate of 2.5% was considered clinically relevant. With an estimated standard deviation of 2% and α = 0.05, we calculated that a sample size of seven individuals per group would be needed to achieve a power of 95%. Therefore, eight individuals per group were included.

TER-alb was calculated and expressed as the percentage disappearance per hour. Fluid filtration capacity (K_f_) was determined by venous occlusion plethysmography in both forearms and averaged. The mean K_f _was used for further calculations. A change in the ratio of ECW/TBW was taken to give an impression of microvascular permeability, using BIA.

Student's t-tests or analysis of variance with repeated measures were used for the assessment of the effects of endotoxin on microvascular permeability parameters. All data are expressed as mean ± standard error of the mean of *n *experiments unless otherwise stated. *P *< 0.05 was considered statistically significant.

## Results

Demographic characteristics of the participants are presented in Table 1. There were no significant differences between the groups.

### Changes in clinical and inflammatory parameters

**Figure 1:**
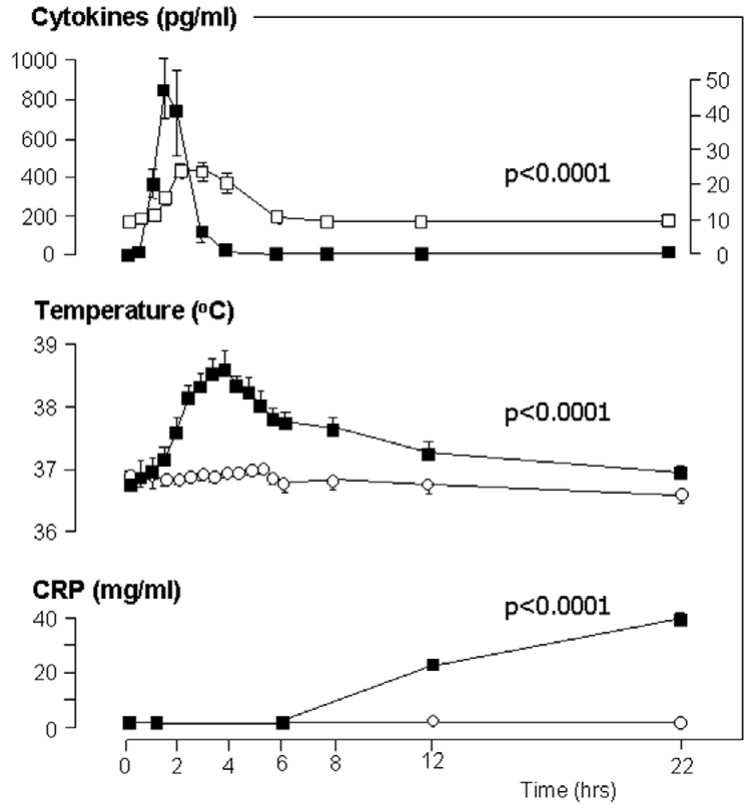
Changes in inflammatory parameters. Administration of endotoxin (*n *= 8; 2 ng/kg) resulted in a marked increase in tumour necrosis factor (TNF)-α (closed squares, left axis) and IL-1β (open squares, right axis). In control individuals cytokine levels remained below the detection limit (*n *= 8; data not shown). Cytokine release was associated with fever and an increase in C-reactive protein (CRP; endotoxin group, closed squares; control group, open circles). Data are expressed as means ± standard error of the mean. The *P *values in the figure refer to the difference between endotoxin and control groups as analyzed using analysis of variance with repeated measures over the complete curve.

The first flu-like symptoms (headache, nausea, chills) occurred in the endotoxin-treated group between 55 and 90 min after LPS injection. Body temperature started to rise 1 hour after endotoxin administration to a maximum of 38.7 ± 0.3°C at 4 hours versus 36.9 ± 0.2°C in the control group (*P *< 0.001). At 8 hours all clinical symptoms had declined to control values. The clinical onset of inflammation was accompanied by a sudden rise in TNF-α plasma levels at 60 min (373 ± 71 pg/ml), which reached its zenith at 90 min (856 ± 158 pg/ml), closely followed by a rise in IL-1β that was maximal at 120 min (23.9 ± 2.2 pg/ml). C-reactive protein increased from under 5 mg/ml at baseline to 22.3 ± 1.4 mg/ml at 12 hours after endotoxin administration and reached its maximum at 22 hours (38.9 ± 3.0 mg/ml). In the control individuals no elevations in temperature (from 36.9 ± 0.1 to 37.0 ± 0.1°C), clinical symptoms, cytokine levels (TNF-α <8 pg/ml, IL-1β <8 pg/ml) or C-reative protein (<5 mg/ml) were observed (Fig. 1).

### Changes in haemodynamic parameters

**Figure 2:**
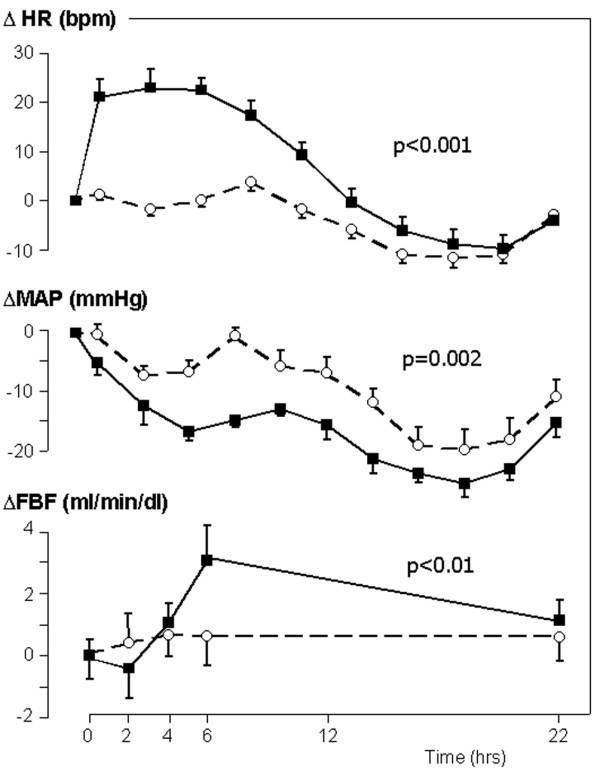
Changes in haemodynamic parameters. Administration of endotoxin (2 ng/kg; *n *= 8; closed squares) resulted in a significant increase in heart rate (HR; measured using electrocardiography; *P *< 0.0001), a significant decrease in mean arterial pressure (MAP; measured intra-arterially; *P *< 0.0001) and a significant increase in forearm blood flow (FBF; measured using venous occlusion plethysmography; *P *= 0.018). HR and FBF did not change significantly in the control group (open circles; *n *= 8), whereas MAP decreased (*P *= 0.035). MAP decreased significantly more in the endotoxin group than in the control group (*P *= 0.002). These changes demonstrate that endotoxin induces a vasodilatory state. Data are expressed as means ± standard error of the mean. The *P *values in the figure refer to the difference between endotoxin and control group as analyzed using analysis of variance with repeated measures over the complete curve.

Figure 2 shows the course of heart rate, mean arterial pressure and forearm blood flow in the endotoxin and control group. In the control group the mean arterial blood pressure decreased from 88 to 80 mmHg at 6 hours (*P *= 0.035); the blood pressure decreased significantly more in the individuals administered LPS (from 96 ± 3 mmHg to 79 ± 4 mmHg at 6 hours, *P *< 0.0001; difference from control individuals: *P *= 0.002). Heart rate remained unchanged in the control group (from 66 ± 4 to 65 ± 2 beats/min; not significant) and increased from 63 ± 3 to 91 ± 3 beats/min at 6 hours in the LPS group (*P *< 0.0001). Forearm blood flow increased from 3.7 ± 0.6 to 6.8 ± 1.1 ml/min per dl at 6 hours (*P *= 0.018) in the endotoxin group, but remained unchanged in the control group (3.8 ± 0.8 versus 4.4 ± 0.9 ml/min per dl; not significant).

### Changes in microvascular permeability parameters

**Figure 3:**
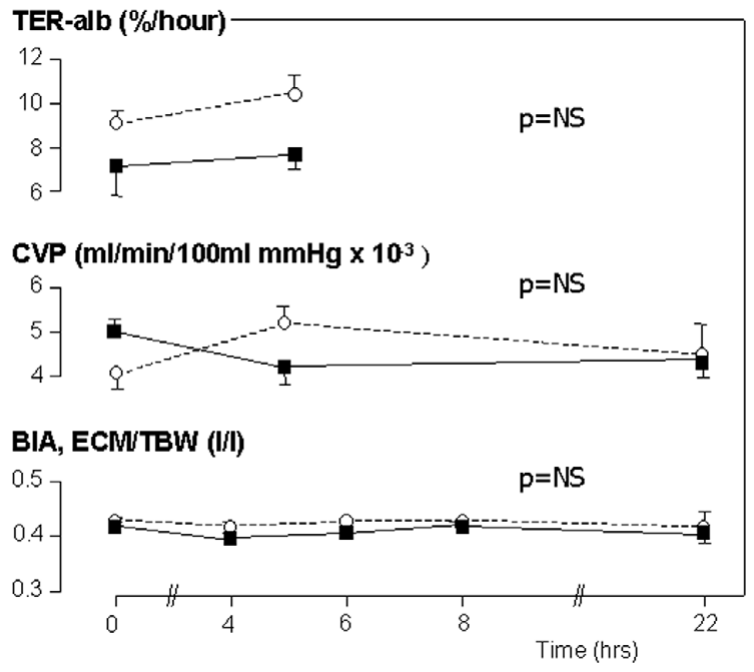
Changes in microvascular permeability parameters. Microvascular permeability parameters were measured using transcapillary escape rate of I^125^-albumin (TER-alb), venous congestion plethysmography (VCP) and bioelectrical impedance analysis (BIA). There were no changes in microvascular permeability as measured using all three parameters in either the endotoxin group (*n *= 8; 2 ng/kg; closed squares) or in the control group (*n *= 8; open circles). Data are expressed as means ± standard error of the mean. ECW, extracellular water; TBW, total body water.

In neither the endotoxin group nor the control group were significant alterations in microvascular permeability parameters detected. In the endotoxin group TER-alb was 7.2 ± 0.6%/hour before and 7.7 ± 0.9%/hour at 4.5 hours after endotoxin administration (not significant); K_f _remained unchanged (from 5.0 ± 0.3 to 4.2 ± 0.4 ml/min per 100 ml mmHg × 10^-3^; not significant); and ECW/TBW, as measured by BIA, did not change (from 0.42 ± 0.01 l/l to 0.40 ± 0.01 l/l; not significant). Also, no significant changes in microvascular permeability parameters were found in the control group (all not significant: TER-alb from 9.08 ± 1.28 to 10.38 ± 0.63%/hour; K_f _from 4.14 ± 0.42 to 5.17 ± 0.39 ml/min per 100 ml mmHg × 10^-3^; and ECW/TBW from 0.43 ± 0.01 l/l to 0.42 ± 0.01 l/l). The effect of endotoxin on microvascular parameters is shown in Fig. 3.

## Discussion

Although administration of endotoxin to human volunteers has facilitated sepsis-associated research, the present study demonstrates that human experimental endotoxaemia is not a suitable model in which to study sepsis-induced changes in microvascular permeability. In a negative study the first issue to address is methodology. We conducted the present study with all three methods that are available for human *in vivo *experiments. Differences in microvascular permeability have been detected in various other diseases with these methods [13-17]. In septic patients an increase in K_f _was demonstrated with TER-alb [18], VCP [19] and BIA [20]. In view of the ability of these methods to detect differences in microvascular permeability and the consistently negative findings of all three methods used in this endotoxin study, we believe our results are valid.

There are several possible reasons for our negative findings. First, the inflammatory stimulus might not have been sufficiently powerful. Endotoxin is known to stimulate the immune system in a dose-dependent manner [25]. Indeed, a marked increase in permeability *in vivo *has previously been shown in, for example, cats after intravenous administration of 1 mg/kg endotoxin [10]. On one occasion, an autointoxication with 1 mg of *Salmonella *endotoxin resulted in profound vasodilatory shock and a 15 l cumulative fluid balance over 72 hours in a laboratory worker [34]. This demonstrates unequivocally that high doses of endotoxin can cause shock and vascular leakage. In human volunteers an endotoxin concentration of 4 ng/kg is considered the maximal tolerable dose. The concentration of 2 ng/kg is widely applied and results in systemic inflammation, activation of coagulation pathways and distinct haemodynamic changes. Although the rise in proinflammatory cytokines is dose dependent, studies that used 4 ng/kg LPS found changes in clinical parameters similar to those reported here (e.g. rise in body temperature and fall in blood pressure) [35]. In the individuals included in the present study (who received 2 ng/kg) the flu-like symptoms, rise in body temperature, rise in heart rate, fall in blood pressure and rise in C-reactive protein were considerable; we therefore believe that the inflammatory stimulus was adequate. Also, the TNF-α and IL-1β concentrations in these individuals exceeded considerably the threshold levels of 50 pg/ml and 20 pg/ml, respectively, that are necessary to increase permeability significantly *in vitro *[6].

Naturally, it remains difficult for many reasons to compare an *in vitro *study in endothelial cells of large vessels with our *in vivo *experiment. The human endotoxaemia model is currently the only available *in vivo *human model that mimics Gram-negative sepsis. Whereas in experimental endotoxaemia the stimulus is restricted to LPS, other (non-LPS) bacterial components are also of importance for the induction of cytokines and the inflammatory response [36] and possibly the induction of vascular leakage. These differences could represent the reason why therapies directed at endotoxaemia itself are not of benefit in patients with septic shock [37]. However, as a model, the changes in haemodynamics that occur during human endotoxaemia are similar to those observed in septic shock, and suggest that endotoxin is a major mediator of the cardiovascular dysfunction that occurs in this condition [35].

A second possible reason for our negative findings is that not only the peak concentration of cytokines but also the duration of the increased level of the inflammatory mediators may be important in the pathophysiology of oedema formation in sepsis. The stimulus caused by a single bolus injection of endotoxin may be too short to induce an increase in microvascular permeability. The induction of capillary leakage *in vitro *was accomplished after incubation with endotoxin or cytokines for 6 hours [6]. Also, in pre-eclampsia a sustained rise in plasma cytokines is associated with an increase in microvascular permeability, suggesting a causal relationship [38]. However, although in some cases of sepsis in humans (e.g. meningococcal disease) elevated serum levels of TNF-α have been found in up to 90% of patients [39], several other clinical studies in septic patients reported only minimally elevated or undetectable levels of TNF-α [40,41]. Because these patients exhibit an overt increase in microvascular permeability, sustained high cytokine levels are apparently not mandatory for the development of oedema.

A third reason is that the timing of the measurements might not have been optimal for the detection of changes in permeability. In previous studies maximal changes in haemodynamic parameters were found between 2 and 6 hours after administration of endotoxin [35]. Because these vascular changes can partly be accounted for by endothelial dysfunction [42], we opted to measure microvascular permeability in the same time window. The possibility that an increase in permeability occurred outside the time window of interest appears unlikely because BIA was unchanged at five time points during the experiment, and K_f _was also unaltered at 22 hours after endotoxin administration. Timing may be of critical importance because an accelerated plasma efflux of albumin was only observed during the early phase of sepsis in rats [43]. Also, late-acting cytokines (e.g. high mobility group protein 1) remain elevated for 16–32 hours after the administration of endotoxin and may play a role in the capillary leak found in septic patients. This and possibly other mediators were not measured during our experiment. Again, BIA and VCP measurements after 22 hours did not reveal an increase in vascular permeability in our experiments, suggesting that a possible late increase in vascular permeability was not missed.

Finally, oedema formation may differ from tissue to tissue and from organ to organ. In human endotoxaemia increases in intestinal permeability [44] and alveolar epithelial permeability [45] were previously demonstrated. In contrast, human endotoxaemia did not induce an increase in the ocular blood–aqueous barrier [46]. With the TER-alb and BIA whole body permeability is assessed, whereas the Filtrass strain gauge plethysmograph focuses on the forearms. An increase in microvascular permeability in, for example, the lungs was not specifically assessed, but if it was present it was insufficient to affect whole body permeability. Administration of iodated albumin as a measure of capillary leak may vary with hydration status, and albumin molecules might be too large to be useful as a sensitive permeability marker. However, these problems are overcome with the use of VCP. We believe that fluid loading would not have altered transcappilary leakage, because with the VCP method a venous occlusion pressure is applied to the forearms, so that vascular permeability is measured independent of the volume status of the subject. The suggestion that permeability might have been increased for smaller molecules than albumin can be ruled out for the same reason.

In summary, we do not believe that the methods used, the timing of the permeability measurements, or the absolute maximal cytokine concentrations can account for the observed lack of effect of endotoxin on microvascular permeability in humans. However, the short duration of cytokine increase possibly played a role.

## Conclusion

Although endotoxin administration to humans has proven to be a valuable model for studying systemic inflammation and coagulation, this model cannot be used to investigate the pathophysiological mechanisms that underlie capillary leakage in sepsis or to evaluate pharmacological interventions aimed at attenuating the increase in microvascular permeability.

## Key messages

• 	Endotoxin administration to humans is a valuable model in which to investigate inflammatory and haemodynamic mechanisms in sepsis.

• 	Endotoxin administration to humans does not affect microvascular permeability measured using TER-alb, VCP and BIA.

• 	Endotoxin administration can not be used as a model to study the pathopysiological mechanisms that underlie capillary leakage in sepsis, or to evaluate the pharmacological interventions aimed at restoring normal microvascular permeability.

## Abbreviations

BIA = bioelectrical impedance analysis; ECW = extracellular water; IL = interleukin; K_f _= filtration capacity; LPS = lipopolysaccharide; TER-alb = transcapillary escape rate of I^125^-albumin; TNF = tumour necrosis factor; TBW = total body water; VCP = venous congestion plethysmography.

## Competing interests

The author(s) declare that they have no competing interests.

## Authors' contributions

LTGJvE (medical student) carried out the experiments, performed the statistical analysis and drafted the manuscript. PP conceived the study, and supervised the experiments and writing of the paper. PS participated in the design of the study and corrected the manuscript. WvdB administrated the Alb125 to the participants and measured the plasma radioactivity. MPWJMB (research nurse) assisted with the coordination and practical conduction of the experiments. JGvdH participated in the design of the study and corrected the manuscript. All authors read and approved the final manuscript.

## Figures and Tables

**Table 1:** Demographic characteristics of the participants

Parameter	Endotoxin group	Control group
*n *(male/female)	8 (4/4)	8 (4/4)
Age (years)	23.9 ± 1.0	22.5 ± 0.8
Length (cm)	176 ± 5	183 ± 3
Weight (kg)	71.8 ± 4.9	70.1 ± 3.6
BMI (kg/m^2^)	23.0 ± 0.7	20.9 ± 0.8
SBP/DBP (mmHg)	127 ± 2/80 ± 3	119 ± 3/73 ± 3
Forearm volume (ml)	1019 ± 95	931 ± 67

Data are expressed as mean ± standard deviation. There were no significant differences between the groups. BMI, body mass index; DBP, diastolic blood pressure; SBP, systolic blood pressure.
